# The Incidence and Breadth of Postoperative Pudendal Nerve Palsy and Its Relationship to Table Traction During Hip Arthroscopy With a Perineal Post

**DOI:** 10.1177/03635465261485701

**Published:** 2026-09-30

**Authors:** Graeme Hoit, Tim Dwyer, Jaskarndip Chahal, Timothy A. Burkhart, Evan Curd, Shgufta Docter, Jordan Farag, John Theodoropoulos, Daniel B. Whelan

**Affiliations:** † Department of Surgery, Division of Orthopaedic Surgery, University of Toronto, Toronto, Ontario, Canada; ‡ Institute of Health Policy, Management and Evaluation, Dalla Lana School of Public Health, University of Toronto, Toronto, Ontario, Canada; § University of Toronto Orthopaedic Sports Medicine, University of Toronto, Toronto, Ontario, Canada; ‖ Division of Orthopaedic Surgery, Women’s College Hospital, Toronto, Ontario, Canada; ¶ Faculty of Kinesiology, University of Toronto, Toronto, Ontario, Canada; # Department of Physiatry and Rehabilitation Medicine, University of Toronto, Toronto, Ontario, Canada; Investigation performed at Women’s College Hospital, Toronto, Ontario, Canada

**Keywords:** femoroacetabular impingement syndrome, hip arthroscopy, postoperative complications, nerve injury

## Abstract

**Background:**

Pudendal nerve palsy (PNP) is a poorly characterized and underreported postoperative complication of hip arthroscopy (HA) with a perineal post. PNP may result in external genital numbness and sexual dysfunction in both men and women.

**Purpose:**

To describe the incidence, breadth, and duration of postoperative PNP symptoms and to evaluate the relationship between PNP and intraoperative limb traction.

**Study Design:**

Cohort study; Level of evidence, 3.

**Methods:**

All patients underwent primary unilateral HA in a supine position with a perineal post. Patients with preexisting PNP symptoms, including sexual dysfunction, were excluded. Intraoperative traction duration and force were recorded using a sensor applied to the perineal post. A PNP screening questionnaire and validated sexual function questionnaires were administered confidentially at 2 weeks, 6 weeks, 3 months, 6 months, and 12 months postoperatively. Patient-reported hip outcomes (International Hip Outcome Tool–33 [iHOT-33]) were completed preoperatively and at 6 and 12 months.

**Results:**

A total of 93 patients were included (mean age, 36.4 years [SD, 9.2 years]; 55% male). The mean traction duration was 80 minutes (SD, 19 minutes). In the acute postoperative period, 77 (83%) patients experienced symptoms consistent with PNP. The most common symptoms were perineal numbness (75%), paresthesia (26%), and pain (18%). Urogenital or sexual dysfunction occurred in 39%, including erectile dysfunction in 22% of men and reduced sexual lubrication in 19% of women. At 3 months postoperatively, 26% of patients continued to have PNP symptoms, with 14% persisting to at least 6 months; all symptoms resolved by 12 months. Patients with PNP had greater traction force (MD, 18.1 kg; 95% CI, 0.3 to 37.1 kg) and longer traction time (MD, 15.4 minutes; 95% CI, 5.5 to 25.3 minutes). Longer traction duration was associated with increased odds of PNP (OR per additional 10 minutes, 1.59; 95% CI, 1.12 to 2.37). Persistence of symptoms beyond 6 weeks was associated with lower iHOT-33 scores at 6 months (MD, −14.4; 95% CI, −24.2 to −4.5).

**Conclusion:**

PNP symptoms were common after HA performed with perineal post traction, but most resolved within 6 months and all by 1 year. Increased traction duration and perineal pressure were significant predictors of symptom development, and persistent dysfunction correlated with lower hip-specific outcome scores.

The use of hip arthroscopy (HA) as a treatment has rapidly increased over the past 2 decades.^15,26^ Primarily, HA has been used in the surgical treatment of young adult hip disorders, such as femoroacetabular impingement, labral tears, and foreign body removal.^23^ The majority of patients undergoing HA are young, active people, between the ages of 18 and 40, with a relatively even male-female ratio.^19,22,32^ HA necessitates distraction of the hip joint for visualization and instrumentation of the joint. This is most commonly achieved with traction on the ipsilateral leg against a perineal post.^7^ Enough traction force is applied to adequately distract the hip to allow for instrumentation during the intra-articular portion of the case, which can range between 45 minutes and 2 hours depending on the nature of the procedure.^2,5,6,13^

A commonly reported but poorly studied complication of HA is pudendal nerve palsy (PNP). The pudendal nerve is responsible for providing sensation to the external genitalia, perineum, and perianal areas in both men and women, motor function to the external urethral sphincter and anal sphincter, and the afferent components of penile and clitoral erection and male ejaculation.^4,18,27,31^ PNP can result in symptoms such as external genital, perianal, and perineum numbness, erectile dysfunction, anorgasmia, and, in rare cases, urinary and fecal incontinence in both men and women.^17,20^ Most affected patients report numbness in the absence of pain, distinguishing the condition from pudendal neuralgia.^28^ The reported rate of PNP in patients with HA has varied widely in the literature, between 1% and 30%.^3,7,10,21,24^

Studies examining PNP post-HA suggest that traction force and duration could be related to the development of PNP.^10,11^ Kocaoglu et al^11^ used a cadaveric model to examine the effect of compressive force on the pudendal nerve from the table traction and determine the main site of compression. Their study found significant compression of the pudendal nerve, particularly on the perineal and dorsal genital branches with the force necessary for hip distraction. However, there is limited information with regard to table traction force and the resultant nerve compression in living patients, and the existing studies do not adequately inquire about the breadth or severity of symptoms patients can have related to PNP. Better characterization of the relationship between table traction force and duration and the development of PNP could help in the future development of prevention strategies.

Therefore, the primary objective of this study was to characterize the incidence, breadth, and duration of PNP after HA with a perineal post. Secondarily, we aimed to establish the relationship between PNP and traction force and time.

## Methods

### Setting and Study Design

The study was a prospective longitudinal observational study of patients with HA at Women’s College Hospital [WCH], an academic ambulatory surgical center in Toronto, Ontario, Canada. All patients were recruited from the outpatient practices of 3 academic orthopaedic staff surgeons (T.D., J.C., D.B.W.) with fellowship training in sports medicine and expertise in HA. The study received ethical approval from the WCH institutional review panel (WCH: 2019-0158-E).

### Patients and Eligibility Criteria

Patients undergoing HA were screened for eligibility by trained research associates and the primary author (G.H.) based on established inclusion and exclusion criteria. Patients were included if they were 18 to 55 years of age, undergoing HA, and proficient in English literacy. Patients were excluded if they had preexisting PNP symptoms, peripheral neuropathy, or sexual dysfunction at baseline; if they were not sexually active; if they had previous hip, genital, or pelvic trauma or surgery; and if they had any pain syndrome diagnoses (eg, fibromyalgia, complex regional pain syndrome).

Eligible patients agreeing to participate in the study provided written consent.

### Data Collection

Relevant patient, clinical, and radiographic data were collected at patient preoperative visits. The International Hip Outcome Tool–33 (iHOT-33), a valid and reliable patient-reported outcome measure for patients undergoing HA,^9,14,29^ was administered preoperatively at baseline. Surgical data, including procedural details, traction force, and traction duration, were collected intraoperatively. PNP screening questionnaires were administered at 2 weeks, 6 weeks, 3 months, 6 months, and 1 year postoperatively. Previously validated sex-specific sexual function questionnaires (Sexual Health Inventory for Men [SHIM]^1^ and modified Female Sexual Function Index [mFSFI]^16^) were administered at 6 weeks, 3 months, 6 months, and 1 year postoperatively. The iHOT-33 questionnaires were administered at 6 and 12 months postoperatively (Figure 1).

**Figure 1.**
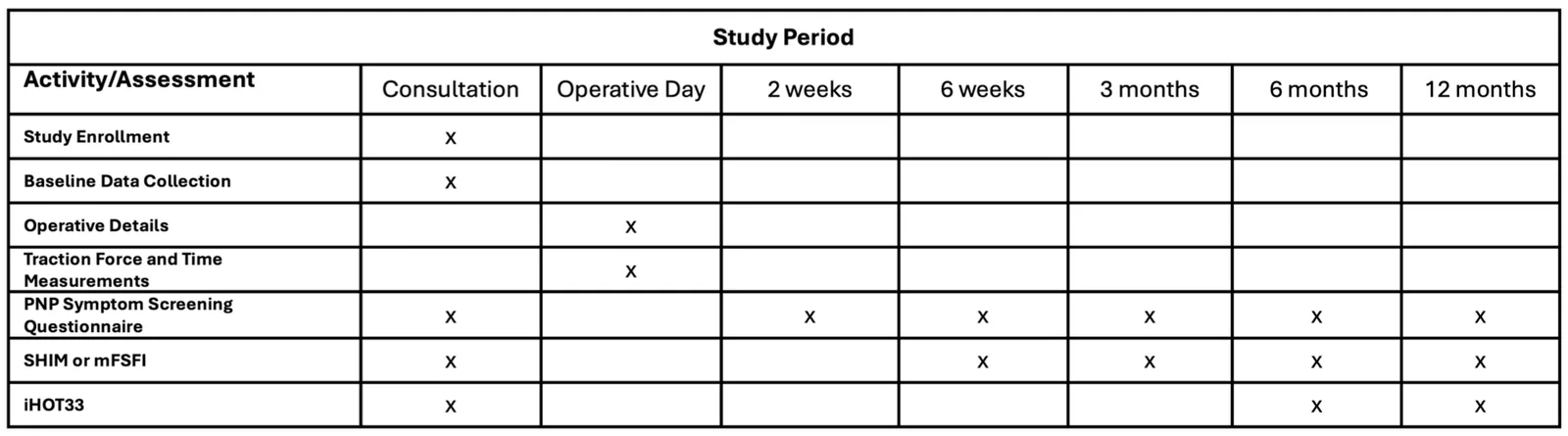
Participant timeline. iHOT33, International hip outcome tool–33; mFSFI, modified Female Sexual Function Index; PNP, pudendal nerve palsy; SHIM, Sexual Health in Men.

### Surgical Procedure and Traction Exposure

Patients underwent HA surgery without alteration to the procedure from the surgeon’s preferred technique. All patients were administered a general anesthetic and positioned supine on a traction table (Smith & Nephew, proprietary operative hip distraction appliance) with a padded perineal post instrumented with a Tekscan FlexiForce 5250 sensor (Figure 2A). Traction was applied under fluoroscopic guidance and titrated to achieve approximately 1 cm of joint distraction, sufficient to permit safe instrumentation during the intra-articular portion of the procedure. The traction time was measured from the time the joint distraction force was applied to the operative limb until it was released, as recorded by the contact pressure sensor and cross-referenced with the operating room record. Traction force was measured by contact pressure mapping from the FlexiForce sensor (Tekscan) (Figure 2B). Surgeons were blinded from intraoperative Tekscan sensor data.

**Figure 2.**
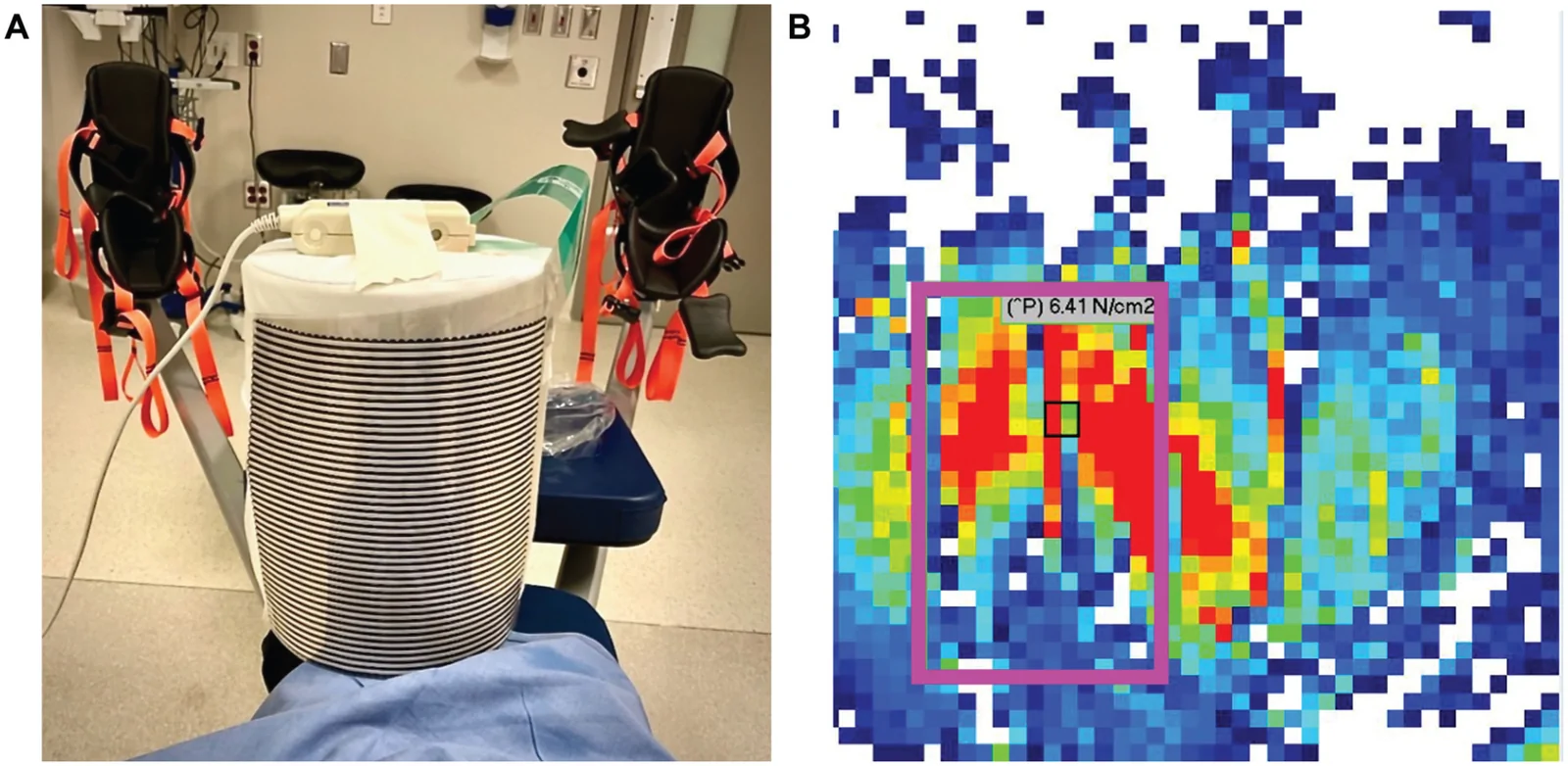
(A) Tekscan force sensor applied to perineal post. (B) Live intraoperative pressure map from force sensors. Red represents areas of increased force, with blue/white showing low/no force on the sensor. The purple box highlights the perineum, with the ischial tuberosities located toward the bottom of the figure.

### Outcomes

The primary outcome was the incidence, breadth, and duration of PNP symptoms, as captured by a PNP screening questionnaire, which was administered at 2 weeks, 6 weeks, 3 months, 6 months, and 12 months postoperatively. The questionnaire inquired about symptoms present at the time of administration, as well as those that were previously present and had since resolved, in an effort to capture symptoms in the early postoperative period. Given that no validated patient-reported PNP questionnaires existed before initiating this study, one was developed for the purpose of the study. Ensuring face validity, an expert panel was assembled with representation from gynecology, urology, peripheral nerve surgery, and clinical medicine, as well orthopaedic surgery, to produce a comprehensive screening questionnaire that was inclusive of all possible PNP symptoms based on known or potential functions of the pudendal nerve. In the development phase, these questions were iteratively given to willing postoperative patients with HA, with feedback on wording and clarity incorporated. The developed questionnaire can be found in Appendix 1. Participants were instructed to answer sexual function questionnaire items based on their current sexual activity, which could include penetrative intercourse, masturbation, and/or oral sexual activity, as applicable to the individual participant and their stage of postoperative recovery. For characterization of postoperative sexual dysfunction, responses were analyzed longitudinally. Participants were considered to have experienced sexual dysfunction if they initially reported engaging in sexual activity and subsequently reported dysfunction on follow-up questionnaires. Sexual dysfunction was considered present from the time of surgery until the first questionnaire on which resolution of symptoms was reported. For example, a participant who reported no sexual activity at the 2-week follow-up and then sexual activity with reduced lubrication at the 6-week follow-up, followed by symptom resolution at the 3-month follow-up, was considered to have experienced sexual dysfunction for a duration of at least 6 weeks.

Secondary outcomes were the iHOT-33 score at 6 months and 1 year postoperatively, as well as sex-specific sexual function patient-reported outcome measures (the SHIM in men and the mFSFI in women) at 6 weeks, 3 months, 6 months, and 1 year postoperatively.

### Analysis of Force and Pressure Sensor Data

The pressure data were imported into the Tekscan analysis software, in which custom regions of interest were developed, to extract data from the whole pressure sensor’s (global) perineum and the thigh. A member of the surgical team (G.H.) visually analyzed each file by identifying standardized landmarks (pubis and ischial tuberosity) at the onset of traction (Figure 2B).

The force, contact area, and contact pressure were exported for each of these regions, in each frame of data, over the duration of the applied traction. The force and pressure data were then imported into a custom LabVIEW program (National Instruments), where the mean and peak pressures were quantified over the duration of the applied traction.

### Statistical Analysis and Sample Calculation

Descriptive statistics were calculated and are presented as mean with standard deviation, mean difference (MD) with 95% confidence interval for continuous variables, where appropriate, and relative frequency with percentage for categorical variables.

Results from the PNP screening questionnaire (primary analysis) were calculated and are described as frequency and proportion at each time point. Patients were classified as having PNP symptoms (yes/no) based on the results of the questionnaire. Patients were classified as having sexual dysfunction (yes/no) based on the sexual symptom subportion of the screening questionnaire.

Associations between traction force and traction time with postoperative symptoms were explored using univariate and multivariable logistic regression modeling. Interactions between traction time and force were explored. Associations between postoperative PNP symptoms and sexual dysfunction with iHOT-33 scores were explored using *t* tests.

All analyses were performed using SAS Version 9.4 (SAS Institute). Statistical significance was set at an alpha value of .05.

### Sample Size Calculation

Given that our primary analysis was descriptive in nature, sample size was calculated on the basis of our most demanding planned analysis—the multivariable logistic regression model for postoperative PNP inclusive of sex, body mass index, traction time, and traction force. Based on an anticipated incidence rate of 50% PNP and the 4 planned covariates in this analysis, we estimated that 80 patients would be needed in our final analysis to avoid overparameterization. Accounting for a 20% sample loss due to dropout, equipment malfunction, or loss to follow-up, we aimed to enroll a final sample size of 96 patients.

## Results

A total of 108 patients consented for enrollment in the study, of whom 102 underwent surgery. Six patients were excluded after consent due to device malfunction, and 3 were either lost to follow-up or withdrew consent for study participation at follow-up, leaving 93 patients in our final study population (Figure 3). The mean age of included patients was 36 years, and 42 (45%) were female (Table 1).

**Figure 3.**
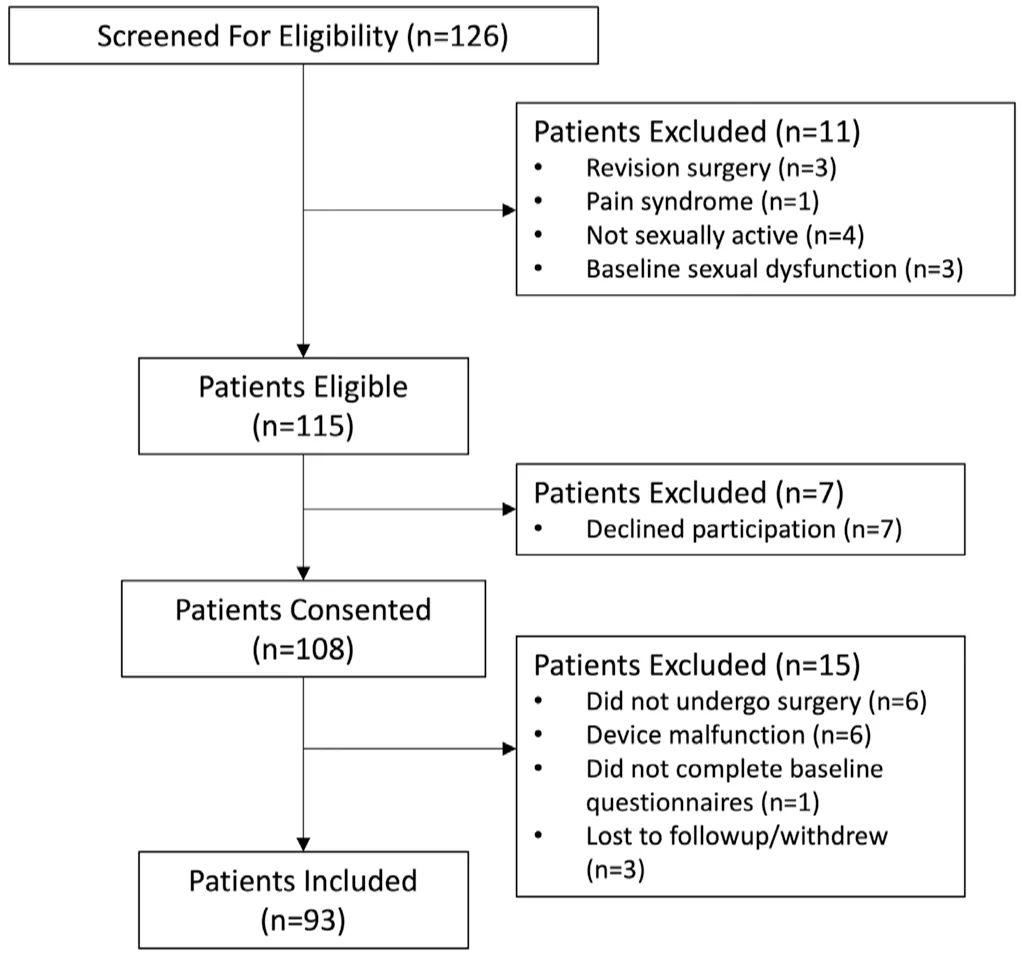
Participant inclusion.

**Table 1:** Patient Characteristics and Operative Details^*a*^

	All Patients	Men	Women
Total No.	93	51 (55)	42 (45)
Characteristics			
Age, y	36.4 (9.2)	35.3 (10.1)	37.6 (8.0)
BMI, kg/m^2^	25.2 (3.6)	25.6 (3.1)	24.7 (4.2)
<18.5	12 (13)	6 (12)	6 (14)
18.5-24.9	39 (42)	20 (39)	19 (45)
25-29.9	34 (37)	22 (43)	12 (29)
30-34.9	7 (8)	3 (6)	4 (10)
35-39.9	1 (1)	00 (0)	1 (2)
≥40	0	0	0
Diagnosis			
Cam	17 (18)	9 (18)	8 (19)
Pincer	7 (8)	4 (8)	3 (7)
Mixed	69 (74)	38 (75)	31 (74)
Patient-reported outcomes			
Baseline iHOT-33 score	32.4 (16.3)	39.0 (16.4)	24.0 (11.7)
Operative details			
Capsular venting	23 (25)	16 (31)	7 (17)
T-capsulotomy	49 (53)	36 (71)	13 (31)
Cam resection	86 (92)	47 (92)	39 (93)
Pincer resection	71 (76)	38 (75)	33 (79)
AIIS resection	38 (41)	24 (47)	14 (33)
OR time, min	112.9 (24.6)	116.0 (25.0)	109.0 (23.8)
Traction details			
Traction time, min	80.4 (19.0)	83.0 (20.2)	77.4 (17.0)
Mean perineal force, kg	56.9 (30.5)	60.4 (34.8)	52.6 (23.8)
Peak perineal force, kg	64.9 (35.2)	69.4 (39.2)	59.2 (28.7)
Mean perineal pressure, kPa	27.6 (11.9)	26.6 (11.5)	28.9 (12.4)
Peak perineal pressure, kPa	34.5 (16.4)	32.8 (15.9)	36.7 (17.0)

a Data are presented as n (%) or mean (SD). AIIS, anterior inferior iliac spine; BMI, body mass index; iHOT-33, International Hip Outcome Tool–33; OR, operating room.

The mean operative time was 112.9 minutes (SD, 24.6 minutes), and the mean traction time was 80.4 minutes (SD, 19 minutes). The mean perineal force, averaged over the total traction time on the perineum, was 56.9 kg (SD, 30.5 kg), and the mean peak force was 64.9 kg (SD, 35.2 kg). For context, the mean perineal force when riding a bicycle is between 38 and 48 kg.^25,30^ Traction forces were higher in male patients than in female patients (Table 1). Patient mean iHOT-33 scores significantly improved from baseline to 6 months (baseline: mean, 32.4 [SD, 16.3]; 6 months: 62.2 [SD, 22.6]; MD, 30.6, 95% CI, 25.3-35.9), and from baseline to 12 months (12 months: mean, 70.5 [SD, 23.5]; MD, 39.2, 95% CI, 33.6-44.9) (Figure 4). At 12 months, 74% of participants met the previously reported iHOT-33 Patient Acceptable Symptom State threshold of 58.^12^

**Figure 4.**
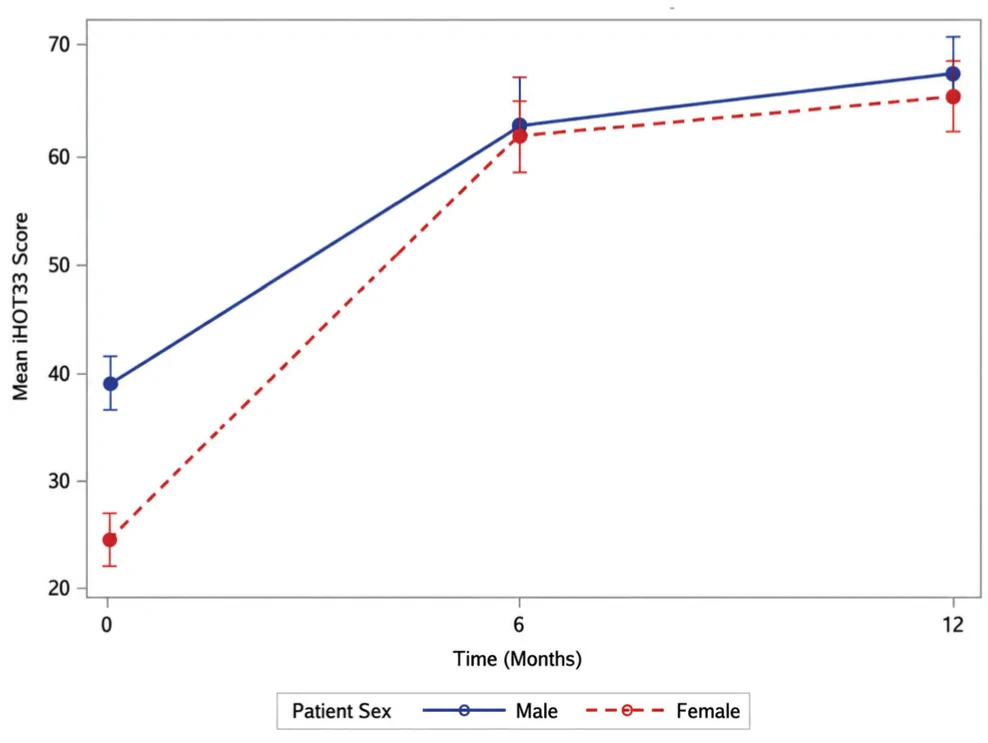
International Hip Outcome Tool–33 (iHOT-33) scores over time stratified by sex.

In terms of our primary outcome, 83% (77/93) of patients experienced PNP symptoms within the initial 2 weeks postoperatively. The most common nerve symptom reported was perineal numbness (70/93; 75%), followed by paresthesia (24/93; 26%) (Table 2). Postoperative urogenital and sexual dysfunction was reported in 39% (36/93) of patients. In men, 11 of 51 (22%) experienced erectile dysfunction. In women, 8 of 42 (19%) experienced decreased sexual lubrication. Anorgasmia was experienced by 22% (20/93) of the patients. Urinary and anal sphincter dysfunction was rare.

**Table 2:** Reported Pudendal Nerve Symptoms Over Time by Sex^*a*^

	Male Patients (n = 51)					Female Patients (n = 42)				
	2 wk	6 wk	3 mo	6 mo	12 mo	2 wk	6 wk	3 mo	6 mo	12 mo
Perineal symptoms										
Any	41 (80)	30 (59)	12 (24)	9 (18)	0	36 (86)	24 (57)	12 (29)	4 (10)	0
Numbness	38 (75)	29 (57)	11 (22)	6 (12)	0	32 (76)	21 (50)	8 (19)	2 (5)	0
Paresthesia	10 (20)	11 (22)	4 (8)	3 (6)	0	14 (33)	9 (21)	4 (10)	2 (5)	0
Itching	10 (20)	4 (8)	2 (4)	3 (6)	0	8 (19)	3 (7)	3 (7)	0	0
Burning	1 (2)	2 (4)	2 (4)	0	0	5 (12)	3 (7)	1 (2)	0	0
Shocks	1 (2)	1 (2)	0	0	0	2 (5)	2 (5)	0	0	0
Heat/cold intolerance	0	1 (2)	0	0	0	0	1 (2)	0	0	0
Perineal pain	7 (14)	6 (12)	2 (4)	1 (2)	0	10 (24)	6 (14)	3 (7)	2 (5)	0
Urogenital function and sexual symptoms										
Any	17 (33)	5 (10)	4 (8)	3 (6)	0	19 (45)	12 (29)	5 (12)	0	0
Erectile dysfunction	11 (22)	1 (2)	3 (6)	2 (4)	0	—	—	—	—	—
Reduced lubrication	—	—	—	—	—	8 (19)	4 (10)	3 (7)	0	0
Anorgasmia	9 (18)	4 (8)	2 (4)	2 (4)	0	11 (26)	8 (19)	3 (7)	0	0
Urinary urgency	1 (2)	1 (2)	1 (2)	0	0	6 (14)	3 (7)	1 (2)	0	0
Urinary incontinence	0	0	0	0	0	5 (12)	3 (7)	1 (2)	0	0
Anal sphincter dysfunction	1 (2)	0	0	0	0	3 (7)	1 (2)	1 (2)	0	0

a Data are presented as n (%). —, not applicable.

The proportion of patients reporting nerve and sexual dysfunction symptoms decreased gradually throughout the 12-month follow-up period. By 6 weeks, 30 (59%) men and 24 (57%) women continued to experience PNP symptoms, including 4 (8%) and 10 (24%) who had persistent sexual dysfunction, respectively (Figure 5). By 6 months, 13 (14%) patients continued to experience nerve symptoms, and 2 (4%) male patients had persistent erectile dysfunction. All symptoms resolved by 1 year (Table 2).

**Figure 5.**
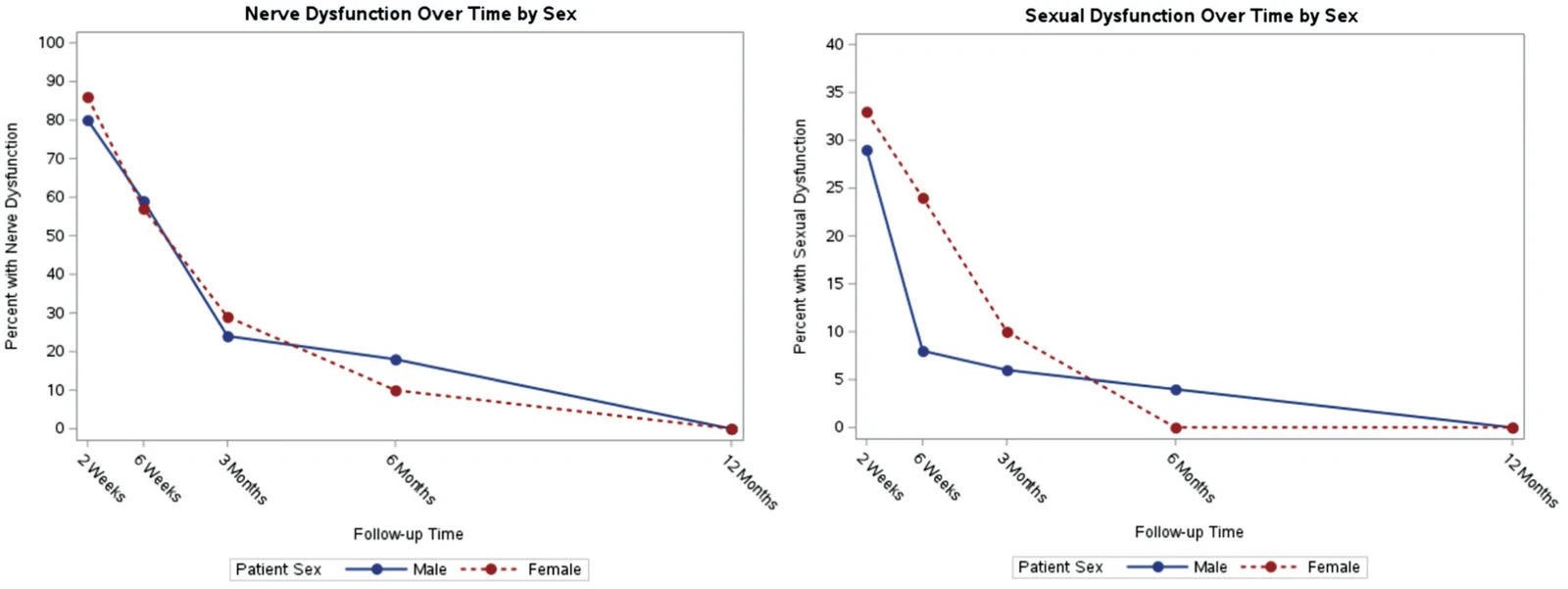
Duration of nerve palsy and sexual dysfunction symptoms.

Patients were significantly more likely to experience nerve symptoms postoperatively with increased traction time (MD, 15.4 minutes; 95% CI, 5.5 to 25.3 minutes), increased peak traction force (MD, 18.1 kg; 95% CI, 0.3 to 37.1 kg), and increased mean traction pressure (MD, 7.4 kPA; 95% CI, 1.0 to 13.8 kPA). Patients were significantly more likely to experience sexual dysfunction symptoms with increased mean traction pressure (MD, 6.5 kPA; 95% CI, 1.3 to 11.7 kPA). Those who had postoperative sexual dysfunction had no significant difference in mean traction time (MD, 6.5 minutes; 95% CI, −1.8 to 14.8 minutes) and traction force (MD, 15.3 kg; 95% CI, −0.6 to 31.2 kg) (Table 3). No significant associations were found between patient-reported sexual function scores and traction variables.

**Table 3A:** Univariate Associations Between Traction Variables and Symptoms^*a*^

Postoperative Outcome	Variable	Symptoms, Mean (SD)	No Symptoms, Mean (SD)	Mean Difference (95% CI)	*P* Value
Nerve symptoms	Traction time, min	83.1 (17.7)	67.7 (20.2)	15.4 (5.5 to 25.3)	.**010**
	Peak traction force, kg	68.2 (35.5)	50.1 (30.2)	18.1 (0.3 to 37.1)	.047
	Mean traction pressure, kPa	28.9 (11.7)	21.5 (11.3)	7.4 (1.0 to 13.8)	.**023**
Sexual dysfunction	Traction time, min	84.9 (18.3)	78.4 (19.1)	6.5 (−1.8 to 14.8)	.12
	Peak traction force, kg	75.5 (36.3)	60.2 (33.9)	15.3 (−0.6 to 31.2)	.06
	Mean traction pressure, kPa	32.1 (11.0)	25.6 (11.8)	6.5 (1.3 to 11.7)	.**015**

a Boldface type indicates statistical significance.

**Table 3B:** Univariate Associations Between Traction Variables and Patient Reported Sexual Function Questionnaires^*a*^

Measure	Variable	*β* Coefficient (95% CI)	*P* Value
SHIM (men)	Traction time	0.09 (−0.04 to 0.22)	.18
	Traction force	0.03 (−0.03 to 0.09)	.32
	Traction pressure	0.15 (−0.06 to 0.37)	.15
mFSFI (women)	Traction time	0.14 (−0.06 to 0.33)	.16
	Traction force	0.000 (−0.12 to 0.11)	.99
	Traction Pressure	−0.02 (−0.30 to 0.26)	.91

a mFSFI, modified Female Sexual Function Index; SHIM, Sexual Health Inventory for Men.

Multivariable modeling demonstrated independent associations between traction time (OR per 10 additional minutes, 1.59; 95% CI, 1.12-2.37) and traction pressure (OR per additional kPA, 1.07; 95% CI, 1.01-1.13) and postoperative PNP (Table 4).

**Table 4:** Multivariable Logistic Regression Assessing Association Variables of Interest and Postoperative Pudendal Nerve Palsy^*a*^

	OR (95% CI)	*P* Value
Traction time, per 10 min	1.59 (1.12-2.37)	.**015**
Traction pressure	1.07 (1.01-1.13)	.**022**
Male sex	0.48 (0.14-1.64)	.24
Age	0.99 (0.92-1.06)	.72
BMI	1.11 (0.92-1.33)	.29
Cam resection	5.96 (0.73-48.42)	.10
Pincer resection	1.74 (0.37-8.29)	.49

a Boldface type indicates statistical significance. BMI, body mass index.

Lastly, the presence of pudendal nerve symptoms was found to be associated with lower 6-month iHOT-33 scores in patients whose symptoms persisted at least 6 weeks (MD, −14.4; 95% CI, −24.2 to −4.5) and at least 6 months (MD, −18.3; 95% CI, −31.4 to −5.2) (Table 5). Similarly, the persistence of sexual dysfunction to 3 months (MD, –18.6; 95% CI, −36.3 to −0.9) and 6 months (−35.6; 95% CI, −67.1 to −4.2) was also associated with lower iHOT-33 scores.

**Table 5:** Association of Postoperative Pudendal Nerve Symptoms With 6-Month Postoperative iHOT-33 Scores^*a*^

Symptom	Duration	iHOT-33 Scores, Mean (SD)		iHOT-33, Mean Difference (95% CI)	*P* Value
		Symptoms	No Symptoms		
Pudendal nerve symptoms	Any	60.5 (23.0)	70.5 (19.0)	−10.0 (−22.5 to 2.5)	.11
	≥6 wk	56.3 (23.3)	70.7 (18.8)	−14.4 (−24.2 to −4.5)	.**005**
	≥3 mo	55.7 (23.8)	64.8 (22.3)	−9.1 (−21.1 to 2.9)	.14
	≥6 mo	46.9 (21.3)	65.2 (21.8)	−18.3 (−31.4 to −5.2)	.**007**
Sexual dysfunction	Any	59.1 (23.7)	63.6 (22.2)	−4.5 (−15.4 to 6.4)	.42
	≥6 wk	55.0 (27.1)	63.6 (21.6)	−8.6 (−22.2 to 5.0)	.21
	≥3 mo	45.6 (29.6)	64.2 (21.6)	−18.6 (−36.3 to −0.9)	.**040**
	≥6 mo	27.4 (20.8)	63.1 (22.0)	−35.6 (−67.1 to −4.2)	.**027**

a Boldface type indicates statistical significance. iHOT-33, International Hip Outcome Tool–33.

## Discussion

In this prospective observational study, postoperative PNP symptoms occurred in 83% of patients undergoing HA performed with perineal post traction. The majority of symptoms were sensory, with perineal numbness most commonly reported, while sexual dysfunction occurred in 39% of patients. Both traction duration and mean perineal pressure were independently associated with the development of PNP, and prolonged traction was related to delayed symptom resolution. Although most symptoms resolved within 6 months, persistent deficits were associated with significantly lower iHOT-33 scores, indicating a measurable impact on hip-specific function and patient-reported outcomes.

The incidence of PNP observed in this study exceeds previously reported rates of 1% to 30%.^3,6-8,10,17,21,24^ There is wide variation in the reported traction times of these studies, with some reporting similar or longer durations than the 80 minutes observed in the present study,^3,6,7,10^ and others reporting shorter traction times.^8,21^ The difference in traction duration may partially explain the variation among these studies, especially given the results of our multivariable model indicating increased odds of PNP with prolonged traction duration. However, given that the incidence of PNP in this study is far beyond the upper limit of the previous reports with similar traction times, variance in traction practice is unlikely to solely explain this discrepancy. Instead, the higher incidence of PNP in this study may reflect the use of a structured, symptom-specific screening tool and prospective follow-up, which together allowed for identification of transient or subclinical symptoms possibly missed in retrospective analyses. The observed relationship between traction parameters and PNP corroborates cadaveric findings by Kocaoglu et al,^11^ who demonstrated significant compression of the pudendal nerve and its branches under traction forces comparable to those used in clinical practice. Our findings extend this work by providing the first in vivo evidence linking increased traction duration and pressure to postoperative PNP in living patients. While prior reports have described PNP as a rare and typically transient complication, the present study quantifies its frequency, recovery timeline, and association with functional outcomes, providing a more comprehensive understanding of its clinical relevance.

Previous literature has failed to adequately characterize pudendal nerve symptoms in the female population, with some studies focusing solely on reporting male sexual symptoms.^3,7^ Sex-specific analysis in this study revealed a high prevalence of pudendal nerve–related symptoms among both men and women, with female patients demonstrating a slightly greater overall symptom burden and a distinct clinical profile. While the overall incidence of perineal symptoms was comparable between sexes (80% of men vs 86% of women), certain symptom categories were more frequently reported by women, including paresthesia (33% vs 20%), burning sensations (12% vs 2%), and perineal pain (24% vs 14%). Notably, female participants also exhibited a higher frequency of urogenital and sexual dysfunction, with nearly half (45%) reporting at least 1 symptom compared with one-third (33%) of men. Despite these sex-based differences in presentation, the temporal pattern of recovery was similar, with the majority of symptoms resolving within 6 weeks and no persistent deficits at 12 months. Importantly, these findings underscore that female-specific symptoms, particularly those related to sexual and urinary function, have likely been underrecognized in prior reports, which have largely lacked validated or targeted screening instruments for women.^10,21^ By using sex-specific measures (SHIM, mFSFI) and direct pudendal symptom assessment, this study provides a more comprehensive characterization of postarthroscopy neuropathy and highlights the need for routine, sex-appropriate evaluation in future research and clinical practice.

The results of this study have important implications for intraoperative practice and patient counseling. Although all PNP symptoms resolved eventually, the high incidence of transient sensory and sexual dysfunction emphasizes the need for patient education regarding potential postoperative effects. Additionally, the persistence of symptoms beyond 3 months in roughly one-quarter of the enrolled patients highlights PNP as a more significant and longer-lasting complication of HA with a perineal post than had previously been reported. The associations identified between traction time, perineal pressure, and PNP support efforts to minimize both variables through careful intraoperative monitoring, the use of wider or more compliant perineal posts, and limiting traction duration whenever possible. Adoption of postless HA techniques may further mitigate pudendal nerve compression and should be explored in future studies. The association between persistent nerve symptoms and lower iHOT-33 scores indicates that even temporary neurapraxia may negatively affect perceived recovery, reinforcing the importance of preventive strategies.

This study has several limitations. First, it was conducted at a single institution, which may limit generalizability to other centers, other traction devices, and other surgical setups. The inclusion of 3 high-volume surgeons does provide some increased generalizability compared to previous single-surgeon series. Second, although the Tekscan sensor provided real-time intraoperative pressure mapping, calibration drift and movement artifacts may have influenced absolute force measurements. Third, the pudendal nerve screening questionnaire, while developed through expert consensus to ensure face validity, has not yet been psychometrically validated, and its performance in detecting mild or transient symptoms requires further study. Additionally, reliance on patient self-report introduces the potential for recall or social desirability bias, particularly for sexual function items. Finally, the absence of a postless control group precludes direct comparison of traction-related risk between different arthroscopic techniques.

## Conclusion

PNP symptoms were common after HA performed with perineal post traction, but most resolved within 6 months and all by 1 year. Increased traction duration and perineal pressure were significant predictors of symptom development, and persistent dysfunction correlated with lower hip-specific outcome scores.
